# Psychosocial Mediation of Light-Moderate Physical Activity and Cognitive Performance among Adults Aged 60+ in China

**DOI:** 10.3390/bs12060175

**Published:** 2022-06-01

**Authors:** Ji Liu, Faying Qiang

**Affiliations:** Faculty of Education, Shaanxi Normal University, Xi’an 710062, China; fayingqiang@163.com

**Keywords:** Chinese older population, physical activity intensity, cognitive performance, psychosocial health

## Abstract

Physical activity is a key determinant of healthy ageing; yet, little is known about the varying degrees of benefits by intensity nor the mediating mechanisms that operate through psychosocial health. Leveraging structural mediation analysis using the 2018 China Health and Retirement Longitudinal Study (CHARLS) national survey data, we screened 4371 community-dwelling older adults, and investigated the mediation mechanism of psychosocial health on the link between light-moderate physical activity and cognitive performance. Physical activity intensity, psychosocial health, and cognitive performance were measured by the international physical activity questionnaire (IPAQ), the Center for Epidemiological Studies Depression Scale (CESD), and the mini-mental state examination (MMSE) instruments, respectively. Results show that, while light physical activity (LPA) and moderate physical activity (MPA) both significantly contribute to better cognitive performance, psychosocial health is a significant mediator only for LPA but not for MPA. For direct pathways, both LPA (std. β = 0.062, *p* < 0.001, 95% confidence interval = 0.032–0.091) and MPA (std. β = 0.049, *p* = 0.001, 95% CI = 0.019–0.078) have significant influence on cognitive performance. For mediation pathways, results show that there exists only one indirect channel through which psychosocial health mediates the influence of LPA (std. β = 0.024, *p* < 0.001, 95% CI = 0.016–0.033), which accounts for 27.9% of the total effect linking LPA and cognitive performance. Findings uncover an important indirect psychosocial mediation channel through which LPA affects cognitive performance among older adults.

## 1. Introduction

Ageing is a continuous and natural deteriorating process associated with impaired physical and mental function, as well as lower quality of life. While life expectancy has gradually increased over recent decades, healthy and disease-free lifespan for the majority remains a global challenge, as longer lives often correlate with increased burden of late-life diseases [1]. Older individuals are especially vulnerable to chronic killer diseases, such as cancer, cardiovascular, and neurodegenerative diseases [2]. Research has indicated that increased risks and onset of Alzheimer’s disease and vascular dementia greatly impact individual’s functional ability, quality of life, and independence at older age [3]. Importantly, cognitive performance has been shown to be a salient determinant of healthy and safe ageing, influencing individual well-being through reducing loneliness [4], improving sleep quality [5], and preventing falls and fall-related injuries [6]. While studies show that behavioral and lifestyle modifications, such as switching to healthy diet [7], increasing exposure to natural environments [8] and social engagement [9], can reduce risks of cognitive impairment among older adults, there is a growing consensus that engagement in frequent physical activity is a leading determinant [10].

At the societal level, many share the view that incentivizing healthy physical behaviors not only lowers individual health risks but also significantly reduces social costs, leading to economically- and socially-desirable outcomes [11]. At the individual level, evidence indicates that promoting physical activity engagement among older adults can be beneficial in combating adverse consequences of age-related decline in cognitive performance. For one, long-term engagement in physical activity can boost the body’s metabolism and reduce risks of obesity, particularly since obesity is a key risk factor for Alzheimer’s disease and vascular dementia in later life [12]. For another, frequent engagement in aerobic exercise can improve glucose regulation, brain processing speed, brain plasticity and blood supply, and can lower the risk of cognitive impairment [13]. To that end, health benefits of frequent physical activity engagement have been widely publicized by the World Health Organization [14].

Yet, while the positive link between physical activity and cognitive performance has been widely acknowledged, little is known regarding the degree of heterogeneity in how such effects vary by physical activity intensity, and their respective channels of physiological and psychological benefits remain under-examined [15]. In specific terms, there exists a stark gap and an ongoing debate in the scientific understanding of the influence of different-intensity aerobic exercises and their benefits and potential repercussions in terms of cognitive impairment and psychosocial factors. For instance, some studies have noted that high-intensity physical activity may exert damaging cognitive effects on some demographic groups, since recent research suggests that frequent and intense exercise may likely increase the risk of rare neurodegenerative disorders [16].

As a consequence of this lack of evidence, most existing physical activity guidelines do not readily differentiate by physical activity intensity. Studies have found that health guidelines tend to only provide information on total activity dose recommendation, while omitting guidance accounting for the multidimensional nature of behavioral engagement that could vary substantially by type and intensity [17]. It was not until recently that the WHO guidelines recommended “150–300 min of moderate-intensity aerobic physical activity; or at least 75–150 min of vigorous-intensity aerobic physical activity”, without fully acknowledging the differential benefits of physical activity at lighter intensity [18].

Evidence on how variation in physical activity intensity is mediated by psychosocial health in affecting cognitive performance is even more scarce. Among a handful of studies that do examine heterogeneous effects by physical activity intensity, little attention is paid to unpacking the direct and indirect mechanisms that operate through psychosocial health and in mitigating risks of cognitive impairment [19]. Prior studies have shown that psychosocial mediation is a critical channel through which physical activity can improve cognitive performance among older adults, in addition to positive physiological change [20]. In this regard, existing evidence suggests that such psychosocial benefits are primarily determined by LPA engagement, which is associated with alleviated release of stress hormones, as well as positive boosts to brain response and nervous system activity [21].

**Figure 1:**
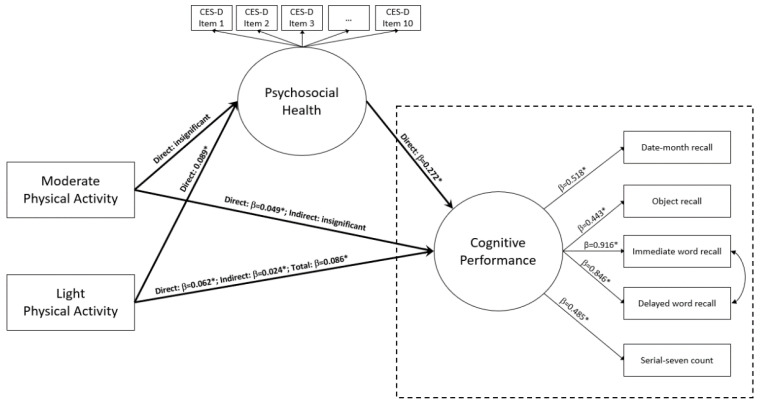
Standardized solution of the structural mediation model. Note: * denotes *p* < 0.05. Model fit indices: χ^2^_135_ = 21426.79 (*p* < 0.001), CFI = 0.905, TLI = 0.889, RMSEA = 0.06, and SRMR = 0.05.

Given the critical multidimensionality of physical engagement for healthy ageing and the lack of understanding with regard to how psychosocial health mediates the cognitive benefits of engaging in physical behavioral modification, the research objectives of this current study are as follows: (1) using a study of a cohort of older adults to examine the heterogeneous relationship between moderate physical activity (MPA, MET values ≈ 4), light physical activity (LPA, MET values ≤ 3.3), and cognitive performance; (2) to assess the degree to which psychosocial health mediates the cognitive benefits of MPA and LPA engagement. In operational terms, this study hypothesizes and builds a structural mediation model to better understand the potential mechanisms underlying the association between activity intensity and cognitive performance in Chinese older adults (Figure 1).

## 2. Methods

### 2.1. Study Participants

This study utilizes a structural mediation model in analyzing the 2018 China Health and Retirement Longitudinal Study (CHARLS), which is a nationally representative survey of adults over the age of 45 in China. The 2018 CHARLS study was conducted between March and September of 2018, consisting of individual interviews of study subjects. The study uses a four-stage (county/village/community/household), stratified (by district/county per capita GDP), cluster proportional-to-size probability sampling, and its core survey modules consist of the following sections: subject demographics, family structure, mental health and physical health assessments, information on health-related behavior and healthcare utilization, work, income, and pension information, etc. The survey response rate is recorded as 83.84% for all surveyed individuals, which is disaggregated at 91.40% in rural areas and 74.55% in urban areas [22].

In the current study, the inclusion of study subjects is determined by the following analytic conditions: (1) completed informed consent to participate; (2) over the age of 60 years old; (3) completed survey modules on physical activity intensity, psychosocial health, and cognitive performance. Conclusively, we screened 4371 subjects who satisfied the inclusion criteria. Research ethics approval for the CHARLS study was granted by the Institutional Review Board at Peking University (IRB00001052-11015). 

### 2.2. Measurement

#### 2.2.1. Measuring MPA and LPA

The CHARLS study gathered background information on subjects’ demographic characteristics, including subjects’ age, sex, cohabitation status, residential status, and educational level, as well as detailed time-use information of subjects’ engagement in various health-related activities. More specifically, the CHARLS study adapted the International Physical Activity Questionnaire (IPAQ) short form to measure subjects’ physically active days per week engaging in MPA or LPA. The IPAQ is an internationally recognized instrument for soliciting and measuring physical activity intensity. In the survey questionnaire, MPA is defined as activities consisting of metabolic equivalent task (MET) values of approximately 4.0, such as “carrying light stuff, bicycling at a normal speed, mopping, Tai-Chi, and speed walking”, whereas LPA is defined as activities involving MET values of less than 3.3, including but not limited to “walking from one place to another, taking a walk for leisure, sports, exercise or entertainment”.

#### 2.2.2. Measuring Psychosocial Health

For psychosocial health measurements, subjects completed a standardized ten-item Center for Epidemiological Studies Depression Scale (CESD) questionnaire, which is an internationally validated instrument for mental health assessment. The CESD has been previously adapted and validated among Chinese older adults and has been shown to demonstrate high sensitivity and accuracy in identification of poor mental health conditions. In the structural mediation model, subjects’ psychosocial health condition is conceptualized as a latent variable, as measured by reverse-scored responses on the ten-item CESD scale, such that higher scores indicate a better state of psychosocial health.

#### 2.2.3. Measuring Cognitive Performance

For cognitive performance measurements, subjects completed a standardized mini-mental state examination (MMSE) questionnaire, which consisted of independent sets of cognitive assessment instruments. The MMSE is a widely used cognition assessment tool designed for older individuals and includes modules assessing orientation, memory, attention and numeracy, recall, and language skills. The translated and locally adapted version of MMSE has been extensively used in China for clinical diagnosis of cognitive impairment and is administered to subjects using their preferred dialect in the CHARLS study. In the structural model, subjects’ cognitive performance is conceptualized as a latent construct measured by scores received on five independently scored tests, namely “Date-month recall”, “Object recall”, “Immediate word recall”, “Delayed word recall”, and “Serial seven count”.

### 2.3. Statistical Analysis

In this study, the analytic procedures unfold in two inter-related steps. In the first analytic step, descriptive information on subjects’ demographics is reported, and bivariate analyses are conducted to assess a series of associations between subjects’ background information and their respective engagement in MPA and LPA. In the second analytic step, we utilize partial least squares structural equation modeling (PLS-SEM) to simultaneously examine the direct relationship between MPA, LPA, and cognitive performance, and fit a mediation model for evaluating the extent to which psychosocial health functions as a mediator for channeling indirect effects in this relationship (see Figure 1). Mediation effects are assessed using three independent statistical tests, including Delta, Sobel, and Monte Carlo, with 5000 bootstrap samples.

Model fit indices, including χ^2^_135_ = 21,426.79 (*p* < 0.001), comparative fit index (CFI = 0.905), Tucker–Lewis index (TLI = 0.889), root mean square error approximation (RMSEA = 0.06), and standardized root mean square residual (SRMR = 0.05), jointly suggest an acceptable model fit. Significance level is set at *p* < 0.05. All statistical analyses were performed in STATA version 15.1 (Stata, StataCorp LLC, College Station, TX, USA) software.

## 3. Results

### 3.1. Participant Demographics

Descriptive information on subjects’ demographic characteristics is itemized in Table 1. For physical activity measures, subjects report engaging in MPA for an average of 2.52 days (SD = 3.10) per week and LPA for 5.41 days (SD = 2.72) per week. Benchmarked against the WHO guidelines [19], the subjects in the sample, on average, tend to be less active than the recommended 3 MPA days per week. Among all 4371 subjects included in the analytic sample, the mean age is reported to be 71.03 years old (SD = 5.16), while 45.66% (*n* = 1966) are female, and 76.92% (*n* = 3362) are married and living with their spouse. For residential status, 23.29% (*n* = 1018) live in urban areas, 6.91% (*n* = 302) live in urban–rural transitional areas, and 69.80% (*n* = 3051) live in rural areas. For educational level, 73.03% (*n* = 3192) of the subjects completed less than lower secondary education, 24.80% (*n* = 1084) completed upper secondary and vocational schooling, and 2.17% (*n* = 95) indicated educational level as “University and above.”

### 3.2. Bivariate Analysis

Columns three to six in Table 1 summarize results from the bivariate analyses between various dimensions of subjects’ demographic variables and MPA and LPA, respectively. In columns three and four, results indicate that there is a statistically significant difference in MPA engagement between males and females (*p* < 0.001) and that MPA engagement varies significantly by residential status (*p* = 0.043). Female subjects tend to report more frequent engagement in MPA than males, whereas subjects living in urban–rural transitional areas tend to report more frequent engagement in MPA than subjects living in urban and rural areas, respectively. There is no discernible difference in MPA engagement by cohabitation status (*p* = 0.565) or educational level (*p* = 0.265). In columns five and six, findings show that there is no statistical difference in LPA engagement by subjects’ sex (*p* = 0.056) nor by cohabitation status (*p* = 0.254). In contrast, significant differences in LPA engagement exist by residential status (*p* < 0.001) and educational level (*p* = 0.001). Subjects living in urban and urban–rural transitional areas report more frequent LPA engagement, whereas subjects with “University and above” education report more frequent LPA engagement than subjects with lower levels of education.

### 3.3. Psychometric Properties of Measurements

In Table 2, the results show the psychometric properties of psychosocial health and cognitive performance instruments utilized in this study. In detail, the information on mean, standard deviation, number of items, Cronbach’s alpha, Kaiser–Meyer–Olkin (KMO) test, and Bartlett’s χ^2^ test statistics is presented. For psychosocial health, the measurements anchor on the standardized ten-item CESD (Mean = 7.71, SD = 1.73), which is evaluated with a Cronbach’s alpha of 0.800, KMO test statistics of 0.883, and Bartlett’s χ^2^ test statistics of 15,040.58 (*p* < 0.05).

With regard to cognitive performance measurements, detailed information on psychometric properties is presented by independent MMSE test components. For the “Date-month recall” test (Mean = 8.02, SD = 2.15), Cronbach’s alpha statistics are reported to be 0.749, whereas KMO and Bartlett’s χ^2^ statistics are 0.859 and 8371.62 (*p* < 0.05), respectively. For the “Object recall” test (Mean = 7.83, SD = 3.00), Cronbach’s alpha is reported to be 0.897, while KMO and Bartlett’s χ^2^ statistics are 0.866 and 5601.52 (*p* < 0.05), respectively. The “Immediate word recall” test (Mean = 3.64, SD = 2.23) is evaluated with a Cronbach’s alpha of 0.870, KMO test statistics of 0.888, and Bartlett’s χ^2^ test statistics of 30,218.61 (*p* < 0.05). For the “Delayed word recall” test (Mean = 3.20, SD = 2.70), Cronbach’s alpha is reported to be 0.796, while KMO and Bartlett’s χ^2^ statistics are 0.887 and 10,368.86 (*p* < 0.05), respectively. For the “Serial-seven count” test (Mean = 3.44, SD = 3.95), Cronbach’s alpha is reported to be 0.914, while KMO and Bartlett’s χ^2^ statistics are reported to be 0.815 and 35,792.82 (*p* < 0.05), respectively. Based on the above results on the instrument psychometric properties, it can be concluded that both measures of psychosocial health and cognitive performance exhibit a generally good level of within-construct validity and reliability.

### 3.4. Structural Mediation Analysis

Following analytic procedures laid out in the statistical analysis plan, this study utilizes PLS-SEM to assess the extent to which psychosocial health (PH) mediates the link between MPA, LPA, and cognitive performance (CP). Table 3 summarizes key results on the direct, indirect, and total effects of the structural mediation model, for which all effects are reported in the form of standardized coefficients (std. β). As previously stated, the structural model is reasonably acceptable, with model fit measures χ^2^_135_ = 21,426.79 (*p* < 0.001), CFI = 0.905, TLI = 0.889, RMSEA = 0.06, and SRMR = 0.05. Generally speaking, the bound limit for CFI and TLI indicating good model fit is 0.90, while for RMSEA and SRMR, their upper-bound limits are 0.06 and 0.08, respectively [23].

For direct pathways, structural results indicate that MPA has significant direct influence on CP (std. β = 0.049, *p* = 0.001, 95% confidence interval, CI = 0.019–0.078) but not on PH (std. β = 0.029, *p* = 0.065). Additionally, model results also show that LPA exhibits significant direct influence on both CP (std. β = 0.062, *p* < 0.001, 95% CI = 0.032–0.091) and PH (std. β = 0.089, *p* < 0.001, 95% CI = 0.058–0.120), while PH has significant direct influence on CP (std. β = 0.272, *p* < 0.001, 95% CI = 0.242–0.303). In relative terms, the significant direct influence on CP is somewhat larger for LPA (std. β = 0.062) as compared to MPA (std. β = 0.049).

In assessing the indirect mediation links, results show that there exists only one indirect channel through which PH mediates the influence of physical activity on cognitive performance, such that LPA has significant indirect influence on CP via PH, but this indirect channel does not exist between MPA and CP via PH. To be clear, results show that there is no statistically significant indirect channel between MPA and CP operating through PH (indirect std. β = 0.008, *p* = 0.067), whereas the indirect link between LPA and CP via PH is statistically significant (indirect std. β = 0.024, *p* < 0.001, 95% CI = 0.016–0.033).

Considering that LPA has a significant direct influence of 0.062 on CP, the indirect influence of 0.024 operating through PH is estimated to be 38.7% of that direct influence (0.024/0.062). To this end, given that LPA has both a direct and indirect channel influencing CP, its total influence on CP is estimated to be 0.086 (*p* < 0.001, 95% CI = 0.055–0.158), of which 27.9% (0.024/0.086) is indirectly channeled through PH.

## 4. Discussion

Motivated by the lack of empirical attention paid to the heterogeneous influence that exists between light-moderate physical activity and cognitive performance and informed by the potential psychosocial mediation on this link, this study hypothesized that MPA and LPA can have heterogeneous regulatory effect on CP, and that PH may act as a key mediator differentiating their cognitive benefits among older populations. We empirically tested this hypothesis utilizing the 2018 CHARLS study, which included 4371 subjects over 60 years old in China.

Based on results from the statistical analyses, a few key findings emerged and are worth highlighting. Firstly, bivariate analyses results show that older adults in China do not systematically distinguish between MPA or LPA in terms of preference for engagement. This result is to be expected, since prior studies have voiced concerns regarding the absence of health guidelines at both national and international levels in making targeted recommendations addressing the multidimensionality of activity type and intensity for healthy ageing [24]. Secondly, PLS-SEM results reveal that while MPA and LPA both exhibit direct influence on CP, their magnitude of influence varies considerably. In detail, the standardized coefficients of MPA’s direct effect on CP is evaluated at 0.049 (*p* = 0.001, 95% CI = 0.019–0.078) compared to that of LPA at 0.062 (*p* < 0.001, 95% CI = 0.032–0.091). This result implies that the direct cognitive benefit of LPA is 26.5% larger than that of MPA (0.062/0.049) among study subjects who are over 60 years old in China. Thirdly, the structural mediation model results uncover new evidence of PH playing a key mediator role in channeling the indirect effects of LPA on CP. In concrete terms, the indirect effect of LPA operating through PH (indirect std. β = 0.024, *p* < 0.001, 95% CI = 0.016–0.033) is estimated to be 38.7% of its direct effect (0.024/0.062) and represents approximately 27.9% of LPA’s total effect on CP (0.024/0.086). Fourthly, the structural mediation model results rule out the possibility of PH mediating indirect effects of MPA on CP, which imply that cognitive effects of MPA are entirely direct and that there is no detectable psychosocial influence on MPA among study subjects who are over 60 years old in China.

The present study is one of few existing studies attempting to partition the influence of MPA and LPA on CP into independently assessed direct and indirect channels, as mediated by PH. By employing a structural mediation analysis, this study contributes new evidence that confirms the existence of substantive heterogeneity in cognitive health benefit channels related to physical activity type and intensity. On the one hand, results in this study corroborate prior research that identified LPA’s positive association with higher levels of domain-specific executive function [25], whereas MPA has been shown to significantly affect memory capacity and working memory performance by strengthening neurogenesis and connectivity of hippocampal neurons [26]. On the other hand, the new findings in this study are consistent with prior research that highlights PH as a key mediator channeling cognitive benefits of physical activity engagement. For one, studies have shown that regular exercise improves PH, which is in turn associated with better CP [27]. For another, research has also suggested that different activity type may have varying degrees of effect in stimulating CP, particularly since lifestyle modulation [28] and social interaction [29] play key roles in determining behavior preference among older populations. In this regard, the existing studies have shown that LPA, as opposed to moderate or vigorous physical activity, tends to be more effective in reducing stress response and in recovering the body’s sympathetic–parasympathetic balance, which can result in improved executive function [30] and enhanced gray matter volume in the brain [31]. These findings may hold potentially strong implications for the design and implementation of comprehensive rehabilitation and combination therapy for older adults [32].

Beyond the contribution of new empirical evidence to the understanding of the multidimensionality of physical engagement for healthy ageing, a key strength in this study is leveraging a large national dataset and employing a PLS-SEM approach, which substantially reduces sampling and measurement bias. By adopting this analytic design, the sampling error is reduced to the extent possible, since CHARLS is nationally representative, whereas the measurement error is minimized, since we conceptualize key outcome and mediator variables as latent constructs comprising vectors of the observed instruments. Additionally, PLS-SEM allows for simultaneity in hypothesis testing, which limits type I error rates that may arise in traditional regression-based analytic designs. Notwithstanding, several limitations to the present study should be well noted in interpreting its findings. First, the non-response and sample attrition rate is non-random across the regions and demographics, which could potentially introduce sampling bias. Second, both the psychosocial health and cognitive performance instruments included items that require self-recall of information, which may become sources for recall bias. Third, it is also key to highlight that, since the present study utilizes cross-sectional data from the 2018 CHARLS dataset, its associational findings are limited in the extent to which causality could be identified. Finally, new evidence presented in this study highlights promising new avenues for further interdisciplinary research in understanding the combined pathophysiological mechanisms of linking physical, psychosocial, and cognitive health, particularly addressing how behavioral modifications through LPA shape individual interaction with psychosomatic stress [33], and in turn, affect mental processes connected to cognition in older populations. 

## 5. Conclusions

In conclusion, this study uncovers a previously under-examined heterogeneous relationship among MPA, LPA, and CP, by illustrating that a key mediation channel exists in linking LPA, PH, and CP. Given that psychosocial and cognitive health benefits vary significantly by physical activity intensity—a key aspect of physical behavioral modifications that has been largely neglected in both health guidelines and previous research—more attention from behavioral scientists is required. In practice, individuals may underestimate the aggregate benefit of LPA, since its net effects on CP are not only channeled directly but also through indirect mediation by PH. To that end, physical activity guidelines and policy guidance by international health authorities, such as the WHO, in promoting healthy ageing should take into consideration the psychosocial mediation on the link between light physical activity and cognitive performance among older adults.

## Figures and Tables

**Table 1:** Demographic characteristics of study subjects by physical activity intensity (*n* = 4371).

	Mean (SD)/ *n* (%)	MPA (Days/Week)		LPA (Days/Week)	
		Mean (SD)	*p*	Mean (SD)	*p*
MPA (days/week)	2.52 (3.10)	-	-	-	-
LPA (days/week)	5.41 (2.72)	-	-	-	-
Age	71.03 (5.16)	-	-	-	-
Sex			<0.001 ^λ^		0.056 ^λ^
Female	1996 (45.66)	2.81 (3.18)		5.32 (2.76)	
Male	2375 (54.34)	2.28 (3.01)		5.48 (2.68)	
Cohabitation status			0.565 ^λ^		0.254 ^λ^
Married and living with spouse	3362 (76.92)	2.54 (3.09)		5.43 (2.71)	
Otherwise	1009 (23.08)	2.47 (3.12)		5.32 (2.77)	
Residential status			0.043 ^δ^		<0.001 ^δ^
Urban	1018 (23.29)	2.59 (3.15)		5.80 (2.38)	
Urban–rural	302 (6.91)	2.92 (3.17)		5.80 (2.46)	
Rural	3051 (69.80)	2.45 (3.37)		5.24 (2.84)	
Educational level			0.265 ^δ^		0.001 ^δ^
Less than lower secondary	3192 (73.03)	2.43 (3.06)		5.37 (2.74)	
Upper secondary and vocational	1084 (24.80)	2.63 (3.12)		5.63 (2.56)	
University and above	95 (2.17)	2.53 (3.12)		5.91 (2.15)	

Note: ^λ^ denotes *t*-test *p* < 0.05, ^δ^ denotes F-test *p* < 0.05.

**Table 2:** Psychometric properties of psychosocial health and cognitive performance (*n* = 4371).

	Mean (SD)	Number of Items	Cronbach’s Alpha	KMO	Bartlett’s χ^2^
Psychosocial health (CESD-10)	7.71 (1.73)	10	0.800	0.883	15,040.58 *
Date-month recall (MMSE-1)	8.02 (2.15)	10	0.749	0.859	8371.62 *
Object recall (MMSE-2)	7.83 (3.00)	16	0.897	0.866	5601.52 *
Immediate word recall (MMSE-3)	3.64 (2.23)	10	0.870	0.888	30,218.61 *
Delayed word recall (MMSE-4)	3.20 (2.70)	10	0.796	0.887	10,368.86 *
Serial-seven count (MMSE-5)	3.44 (3.95)	5	0.914	0.815	35,792.82 *

Note: * denotes *p* < 0.05.

**Table 3:** Results from the structural mediation model (*n* = 4371).

Regression Pathways	Std. β	95% CI	*Z*	*p*
Direct effects				
MPA → CP	0.049 *	(0.019, 0.078)	3.24	0.001 *
MPA → PH	0.029	(−0.002, 0.060)	1.84	0.065
LPA → CP	0.062 *	(0.032, 0.091)	4.12	<0.001 *
LPA → PH	0.089 *	(0.058, 0.120)	5.68	<0.001 *
PH → CP	0.272 *	(0.242, 0.303)	17.40	<0.001 *
Indirect effects				
MPA → PH → CP	0.008	(−0.002, 0.016)	1.84	0.067
LPA → PH → CP	0.024 *	(0.016, 0.033)	5.41	<0.001 *
Total effect				
LPA → CP (direct + indirect)	0.086 *	(0.055, 0.158)	5.60	<0.001 *
Cognitive performance ~				
Date-month recall	0.518 *	(0.496, 0.541)	45.45	<0.001 *
Object recall	0.443 *	(0.419, 0.467)	36.32	<0.001 *
Immediate word recall	0.916 *	(0.904, 0.928)	150.44	<0.001 *
Delayed word recall	0.846 *	(0.834, 0.857)	142.78	<0.001 *
Serial-seven count	0.485 *	(0.462, 0.508)	41.65	<0.001 *

Note: * denotes *p* < 0.05.

## Data Availability

Restrictions apply to the availability of data used. Deidentified participant data is available upon reasonable request to the corresponding author, with permission of the CHARLS group at Peking University.
